# Association of sodium intake with diabetes in adults without hypertension: evidence from the National Health and Nutrition Examination Survey 2009–2018

**DOI:** 10.3389/fpubh.2023.1118364

**Published:** 2023-08-31

**Authors:** Li Ming, Duan Wang, Yong Zhu

**Affiliations:** ^1^Department of Pediatrics, Xinqiao Hospital, Army Medical University, Chongqing, China; ^2^Department of Rehabilitation, Children’s Hospital of Chongqing Medical University, Chongqing, China; ^3^Department of Pediatric Intensive Care Medicine, Zhangzhou Affiliated Hospital of Fujian Medical University, Fujian, China

**Keywords:** sodium intake, diabetes, NHANES, diet, non-hypertension

## Abstract

**Background:**

Sodium is essential for human health, however the prevalence of various diseases is associated with excessive sodium intake, particularly cardiovascular disorders. However, in most countries, salt intake is much higher than the World Health Organization recommends. Several studies in recent years have revealed that high salt intake is associated with diabetes in the general population, but the association is uncertain in people who do not have hypertension. In this study, we aimed to find out whether high sodium intake increases the risk of diabetes in this particular population.

**Method:**

Data were extracted from the National Health and Nutrition Examination Survey (NHANES; 2009–2018). Participants included adults aged over 20 years old who have undergone the diabetes questionnaire, and the hypertension population was excluded. In order to adjust the confounders, multivariate analysis models were built. Finally, subgroup analysis were conducted to investigate the association between sodium intake and diabetes separately.

**Result:**

In the present study, 7,907 participants are included (3,920 female and 3,987 male), and 512 (6.48%) individuals reported diabetes. The median sodium intake of the participants was 3,341 mg/d (IQR: 2498, 4,364 mg/d). A linear association between sodium intake and the prevalence of diabetes was found (*p* = 0.003). According to the multivariate analysis models, the odds ratio of diabetes for every 1,000 mg sodium intake increment is 1.20 (OR: 1.20, 95% CI 1.07–1.35). The highest sodium intake quartile was 1.80-fold more likely to have diabetes than the lowest quartile (OR: 1.80, 95% CI 1.17–2.76).

**Conclusion:**

Our results suggest that higher sodium intake is associated with an increased risk of diabetes in the population without hypertension, and for every 1,000 mg sodium intake increment, the risk of diabetes increased by 1.20-fold. To sum up, we have provided the clue to the etiology of diabetes and further prospective research is needed to contribute recommendations for the primary prevention of diabetes in the US.

## Introduction

Sodium is a physiologically essential nutrient to maintain human health and well-being. As an osmotic determinant of extracellular fluid and plasma volume, it has a variety of functions in the body, including regulating nerve and muscle function and maintaining acid–base and water balance (1, 2). It has been found that excessive intake of sodium is linked to the increased risk of various chronic diseases, especially cardiovascular disease (3–5). However, the relationship between sodium intake and diabetes remains unknown.

Over 90% of the sodium in the diet comes from table salt, while processed foods account for 75% of salt consumption (6). The World Health Organization (WHO) proposes to reduce sodium intake to less than 2000 mg/day in the general population (7). However, in most countries, sodium consumption is significantly higher than the recommended amount. In 2010, the global average sodium intake was 3.95 g per day, which is equivalent to 10.06 g of salt per day and nearly twice the WHO recommended limit of 2 g per day (8). The most recent update of the National Academy’s Dietary Reference Intakes for Sodium and Potassium (2019) recommends all adults 19 years of age and older have a sodium adequate intake of 1,500 mg/d (9). In the United States, about 90% of adults consume sodium more than the recommended amount with an average of 3,400 milligrams per day (10). Excessive sodium intake is also prevalent in Asian countries. In China, about 92.6% of adults consume more sodium than the recommended amount, with an average of 5,013 milligrams per day (11). Various countries, both developed and developing, are adopting strategies to decrease the consumption of salt among their populations. These strategies encompass the establishment of salt targets for food, enforcing mandatory labeling for high-salt products, and educating consumers.

More than 34 million people in the United States have diabetes, which has a huge impact on public health globally (12) and dramatically raises the risk of cardiovascular events, microvascular disease and early death (13). Diabetes places a significant burden on society in the form of increased medical costs, lost productivity, premature mortality, and intangible costs such as decreased quality of life (14). Currently, smoking, high-risk drinking, dyslipidemia, hypertension, and metabolic syndrome were reported as the risk factors for diabetes (15). However, a few studies have explored the relationship between salt and sodium intake and diabetes, and the results have shown inconsistency (16–18), and no studies have looked at the relationship between sodium intake and diabetes in a specific group of people without hypertension. Thus, the relationship between sodium intake and diabetes is needed to further probe.

The National Health and Nutrition Examination Survey (NHANES) is a research program designed to assess the health and nutrition status of adults and children in the United States. It combines interviews and physical exams, focuses on a variety of health and nutrition measures, and provides a range of data, including socioeconomic, diet, health status, demographics, and laboratory tests (19). In this study, we analyzed data from the 2009 to 2018 National Health and Nutrition Examination Survey (NHANES) to examine the association between sodium intake and diabetes in US adults. Aim to find out whether high sodium intake increased the risk of diabetes.

## Materials and methods

### Study design and population

The NHANES is a series of cross-sectional, complex, multistage surveys conducted by the Centers for Disease Control (CDC) and Prevention of a nationally representative group of non-institutionalized members of the US population designed to provide health and nutrition data (20). Before taking part, participants provided written informed consent. The NCHS ethics review board reviewed and approved the collection of NHANES data (21).

**Figure 1:**
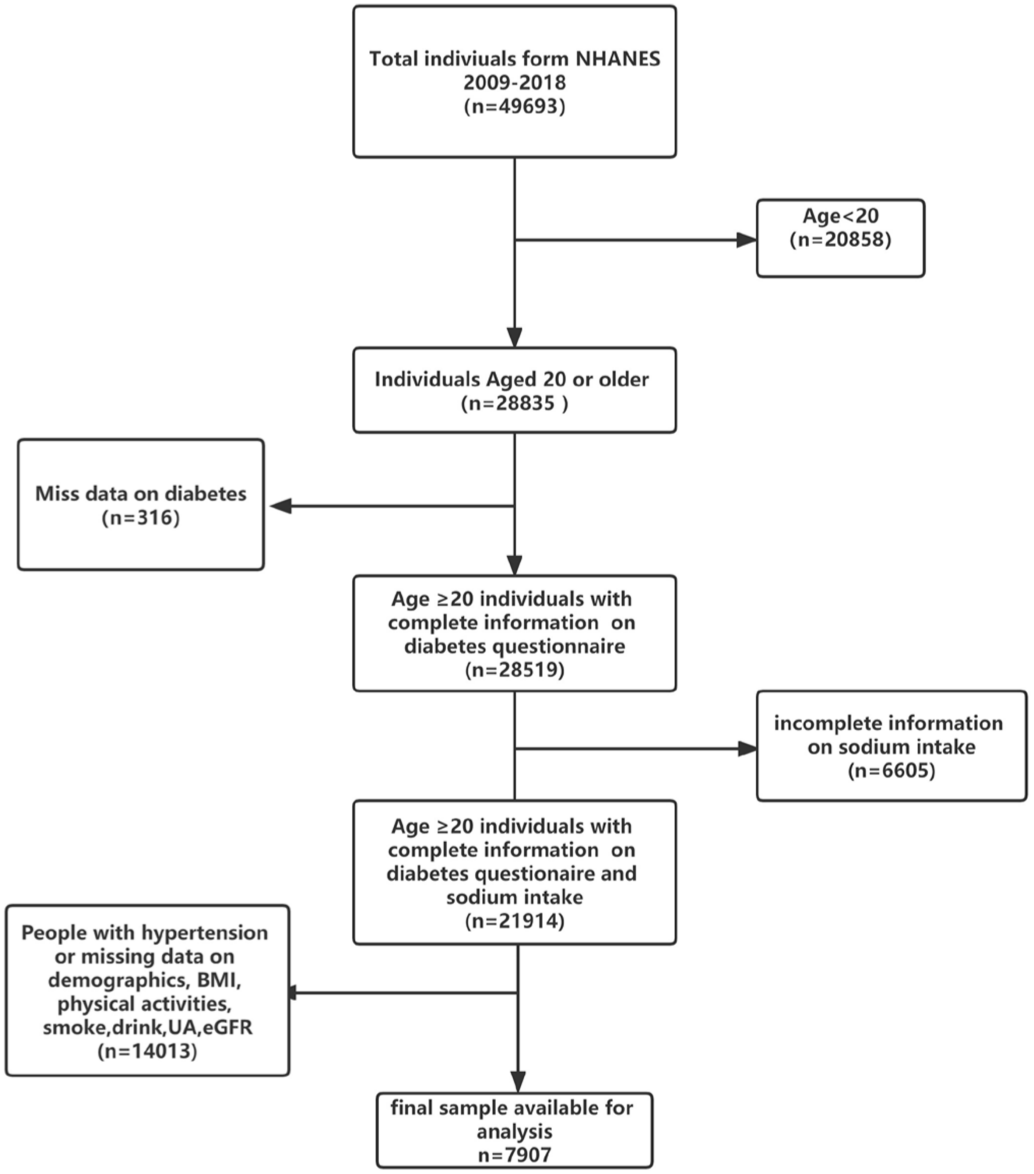
Flowchart of the sample selection from NHANES 2009–2018.

In the present study, we have analyzed the participants from 5 cycles of NHANES (2009–2018). A total of 28,835 adults aged over 20 years old were enrolled. We excluded 316 participants who had missing data on the diabetes questionnaire, and 6,605 participants who had missing sodium intake, 8,899 participants who had missing demographic data and other covariates data, people with high blood pressure (n = 5,114). Finally, 7,907 participants were included in our study (Figure 1) Referring to other previous studies and NHANES official recommendations, we divided the population into three groups according to age (22, 23), among them, 4,016 young adults (ages 20–39 years), 2,720 middle-aged adults (age 40–59 years), and 1,171 older adults (age 60 and older). Meanwhile, fasting blood glucose (FBG, n = 3,825) and glycohemoglobin (HbA1c, n = 7,893) data of the final population were collected as outcome variables to further verify the correlation between sodium intake and diabetes.

### Dietary sodium intake

Dietary sodium intake (mg/day) was the main exposure of interest, face-to-face interviews between trained interviewers and respondents were used to collect dietary intake data form participants. Respondents were asked to recall all foods and beverages consumed in the previous 24 h. All participants were asked to participate in two 24-h total nutrient recall interviews, the second of which was conducted over the phone 3 to 10 days later. As a result, if an individual performed two 24-h recalls, the average sodium intake of the two 24-h recalls was used. Otherwise, we use statistics from the first 24-h recall.

### Definition of outcome variable and hypertension

Diabetes was defined as being informed by a doctor/health professional about the diagnosis of diabetes, and/or the use of insulin or oral hypoglycemic medication (24). According to the diagnostic criteria for diabetes, the outcome variable FBG was divided into groups based on whether it was ≥ 7.0 mmol/L, and glycosylated hemoglobin was divided into groups based on whether it was ≥ 6.5%. Hypertension was defined as a mean SBP ≥ 130 mm Hg, or a mean DBP ≥ 80 mm Hg, or was taking hypertension medication or was informed of a hypertension diagnosis by a physician/health professional.

### Potential covariates

Demographic variables include age (classified as 20–39 years, 40–59 years, and ≥ 60 years), gender, race (non-Hispanic white, non-Hispanic black, Mexican American, other Hispanic, or others), levels of education (below high school, high school and above high school), poverty income ratio (<1.35, 1.35–1.85, >1.85). Body mass index (BMI) is categorized as under/normal weight (<25 kg/m2), overweight (25–29.9 kg/m2), and obese (≥30 kg/m2). Serum cotinine level is used as a marker for smoke exposure (<0.05 ng/mL: unexposed or non-smoker; 0.05–10 ng/mL: Passive smokers; >10 ng/mL: active smoker) (25, 26). Drinking status is classified as never (any year < 12 cups), former (≥12 cups in any year, not drinking now), and current (≥12 cups in any year, currently drinking). The level of physical activity is measured by the MET-min/week, low (500 MET-min/wk), moderate (500–2,999 MET-min/wk), and high (>3,000 MET-min/wk) exercise were the three categories used to categorize total activity (27). Other covariates include total energy intake (Kcal/d), protein (g/d), carbohydrate (g/d), fat (g/d), uric Acid (UA, mg/dL), estimated glomerular filtration rate (eGFR, mL/min/1.73m^2^).

### Statistical analysis

Continuous variables were compared using the one-way ANOVA (normal distribution), Kruscal Whallis H (skewed distribution) among the different groups. Categorical variables were compared using the chi-squared test among the different groups. Descriptive statistics were used to characterize the participants across the quartiles of sodium intake, continuous variables were expressed as mean ± standard deviation, categorical variables were expressed in frequency and percentage. Multivariate logistic regression analysis was used to evaluate the blood pressure based on sodium intake (continuous and categorical variables). Model I adjusted for: age, sex, protein, carbohydrate, and fat. Model II adjusted for: Model I + race, poverty income ratio, BMI, and education. Model III adjusted for: Model II + UA, alcohol intake, eGFR, smoke exposure, and total physical activity. Tests for trend (*P* for trend) were performed by entering the sodium intake group as a continuous variable and rerunning the corresponding regression models. In addition, a generalized additive model and fitted smoothing curve were used to characterize the shape of the relationship between sodium intake and.outcome variable Finally, we also conducted subgroup analysis, investigating the association between sodium intake and diabetes in the subgroup separately. Although some studies have suggested using NHANES sample weights in the analysis to obtain unbiased national estimates, this has the limitation of reducing the statistical power of the study. To balance this relationship, we included sample weights in the sensitivity analysis of the primary outcome variables.

All of the analyses were performed with the statistical software packages R (http://www.R-project.org, The R Foundation) and EmpowerStats (http://www.empowerstats.com, X&Y Solutions, Inc., Boston, MA). *p* values <0.05 (two-sided) were considered statistically significant.

## Results

In the present study, 7,907 participants are included in our study (3,920 female and 3,987 male). Among them, 512 (6.48%) individuals reported diabetes. The median sodium intake of the participants was 3,341 mg/d (IQR: 2498, 4,364 mg/d).

### Demographic characteristics of participants

Table 1 shows the baseline characteristics of participants by quartiles of daily dietary sodium intake. The youth have a higher sodium intake (58.1% vs. 45.1%). Compared to female, the male has a huge percentage of high sodium intake (28.9% vs. 78.4%). For dietary intake, the individuals with higher sodium intake consumed more energy intake (3079.7 ± 858.1 vs. 1379.9 ± 419.7 Kcal), protein (124.3 ± 39.2 vs. 52.5 ± 19.4 g), carbohydrate (357.5 ± 117.3 vs. 177.9 ± 65.3 g) and fat (121.5 ± 42.2 vs. 48.4 ± 19.9 g). In terms of lifestyle, participants who have a higher sodium intake are also more active in total physical activity (39.2% vs. 53.8%) and smoking (23.4% vs. 28.2%) and drinking (73.6% vs. 81.9%). And we found that uric acid level in the high sodium intake population significantly rose (5.0 ± 1.3 vs. 5.5 ± 1.3) too. In addition, people with lower education levels, PIR and eGFR have lower salt intake.

**Table 1:** Characteristics of participants by quartiles of sodium intake in the 2009–2018 continuous NHANES.

Characteristics	Sodium Quartiles				*value of p*
	Quartile 1	Quartile 2	Quartile 3	Quartile 4	
**No of participants**	1976	1977	1977	1977	
**Age (year)**					<0.001
20–39	891 (45.1%)	950 (48.1%)	1,027 (51.9%)	1,148 (58.1%)	
40–59	720 (36.4%)	700 (35.4%)	660 (33.4%)	640 (32.4%)	
> = 60	365 (18.5%)	327 (16.5%)	290 (14.7%)	189 (9.6%)	
**Sex**					<0.001
Female	1,404 (71.1%)	1,211 (61.3%)	877 (44.4%)	428 (21.6%)	
Male	572 (28.9%)	766 (38.7%)	1,100 (55.6%)	1,549 (78.4%)	
**Race**					<0.001
Mexican American	291 (14.7%)	284 (14.4%)	281 (14.2%)	301 (15.2%)	
Non-Hispanic Black	388 (19.6%)	311 (15.7%)	292 (14.8%)	303 (15.3%)	
Non-Hispanic White	811 (41.0%)	925 (46.8%)	938 (47.4%)	887 (44.9%)	
Other Hispanic	241 (12.2%)	174 (8.8%)	189 (9.6%)	162 (8.2%)	
Other Race - Including Multi-Racial	245 (12.4%)	283 (14.3%)	277 (14.0%)	324 (16.4%)	
**Educational level**					<0.001
Below high school	377 (19.1%)	296 (15.0%)	249 (12.6%)	272 (13.8%)	
High school	417 (21.1%)	362 (18.3%)	371 (18.8%)	461 (23.3%)	
Above high school	1,182 (59.8%)	1,319 (66.7%)	1,357 (68.6%)	1,244 (62.9%)	
**PIR**					<0.001
<1.35	660 (33.4%)	524 (26.5%)	584 (29.5%)	595 (30.1%)	
1.35–1.85	250 (12.7%)	235 (11.9%)	191 (9.7%)	221 (11.2%)	
>1.85	1,066 (53.9%)	1,218 (61.6%)	1,202 (60.8%)	1,161 (58.7%)	
**BMI(kg/m2)**					0.036
Under/normal weight	693 (35.1%)	728 (36.8%)	707 (35.8%)	733 (37.1%)	
Overweight	658 (33.3%)	636 (32.2%)	641 (32.4%)	701 (35.5%)	
Obese	625 (31.6%)	613 (31.0%)	629 (31.8%)	543 (27.5%)	
**Energy (Kcal)**	1379.9 ± 419.7	1854.0 ± 421.5	2262.2 ± 481.0	3079.7 ± 858.1	<0.001
**Protein, g**	52.5 ± 19.4	72.6 ± 18.3	90.0 ± 22.5	124.3 ± 39.2	<0.001
**Carbohydrate, g**	177.9 ± 65.3	227.4 ± 71.5	270.4 ± 78.8	357.5 ± 117.3	<0.001
**Fat, g**	48.4 ± 19.9	69.9 ± 21.7	86.5 ± 24.9	121.5 ± 42.2	<0.001
**UA, mg/dl**	5.0 ± 1.3	5.1 ± 1.3	5.3 ± 1.3	5.5 ± 1.3	<0.001
**eGFR, mL/min/1.73 m2**					0.002
<60	53 (2.7%)	33 (1.7%)	30 (1.5%)	28 (1.4%)	
60–89	485 (24.5%)	501 (25.3%)	491 (24.8%)	426 (21.5%)	
≥90	1,438 (72.8%)	1,443 (73.0%)	1,456 (73.6%)	1,523 (77.0%)	
**Alcohol intake**					<0.001
Never	327 (16.5%)	235 (11.9%)	200 (10.1%)	146 (7.4%)	
Former	195 (9.9%)	201 (10.2%)	184 (9.3%)	212 (10.7%)	
Current	1,454 (73.6%)	1,541 (77.9%)	1,593 (80.6%)	1,619 (81.9%)	
**Smoke exposure**					<0.001
Unexposed or non-smoker	1,054 (53.3%)	1,107 (56.0%)	1,040 (52.6%)	924 (46.7%)	
Passive smokers	459 (23.2%)	441 (22.3%)	485 (24.5%)	496 (25.1%)	
Active smoker	463 (23.4%)	429 (21.7%)	452 (22.9%)	557 (28.2%)	
**Total physical activity (MET-min/week)**					<0.001
Low (<500)	342 (17.3%)	307 (15.5%)	270 (13.7%)	200 (10.1%)	
Moderate (500–2,999)	860 (43.5%)	923 (46.7%)	812 (41.1%)	713 (36.1%)	
High (>3,000)	774 (39.2%)	747 (37.8%)	895 (45.3%)	1,064 (53.8%)	
**Diabetes**					0.520
No	1842 (93.2%)	1840 (93.1%)	1852 (93.7%)	1861 (94.1%)	
Yes	134 (6.8%)	137 (6.9%)	125 (6.3%)	116 (5.9%)	
**HbA1c**					0.963
<6.5%	1876 (95.28%)	1886 (95.54%)	1881 (95.24%)	1881 (95.24%)	
≥6.5%	93 (4.72%)	88 (4.46%)	94 (4.76%)	94 (4.76%)	
**FBG, mmol/L**					0.517
<7.0	898 (94.9%)	929 (93.7%)	901 (94.6%)	876 (93.6%)	
≥7.0	48 (5.1%)	62 (6.3%)	51 (5.4%)	60 (6.4%)	

Values are means ± SD or n (%) SD, standard deviation. PIR, poverty income ratio; BMI, Body mass index; UA, Uric Acid; eGFR: estimated glomerular filtration rate; HbA1c: glycosylated hemoglobin; FBG: fasting blood glucose.

### Relationship between sodium intake and diabetes, FBG, and HbA1c

**Figure 2:**
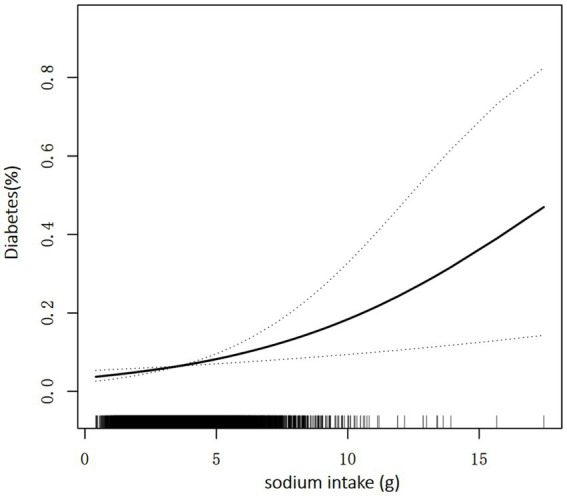
Association between sodium intake and the prevalence of diabetes. A linear association between sodium intake and the prevalence of diabetes was found (*p* = 0.003). The solid line and dashed line represent the estimated values and their corresponding 95% confidence interval. Adjustment factors included age, sex, race, poverty income ratio, BMI, education, energy, protein, carbohydrate, fat, UA, alcohol intake, eGFR, smoke exposure, and total physical activity.

Table 2 shows the relationship between sodium intake and diabetes through multivariate analyses in the included participants. After adjustment for age, sex, energy, protein, carbohydrate, and fat, there is a significant positive correlation between sodium intake and prevalence of diabetes, similar results are found after further adjustment for the other covariates in Table 1. As shown in Table 2, every 1,000 mg sodium intake increment had a multivariate-adjusted odd ratio (OR) with a 95% confidence interval (CI) of 1.20 (95% CI 1.07–1.35) (*p* = 0.003) for diabetes. When continuous sodium intake was converted to quartiles of sodium intake, we found that compared to those in quartile 1 (sodium intake <2,498 mg/d), the adjusted ORs for participants in quartile 2 (2,498 ≤ sodium intake <3,341 mg/d), quartile 3 (3,341 ≤ sodium intake <4,364 mg/d) and quartile 4 (sodium intake ≥ 4,364 mg/d) were 1.30 (95% CI 0.98–1.72), 1.32 (95% CI 0.96–1.83) and 1.80 (95% CI 1.17–2.76) (*p* = 0.008), respectively. There was a trend for higher OR of diabetes among participants in the higher quartile of sodium intake relative to the lower quartile (p for trend =0.016). As shown in Supplementary Tables S1, S2, similar results were observed for the association between salt intake and FBG and HBA1c, every 1,000 mg increase in sodium intake, the multivariate adjusted ratio (OR) for FBG ≥ 7 mmol/L was 1.31 (95% CI 1.14–1.49) (*p* < 0.001), and every 1,000 mg increase in sodium intake, the multivariate adjusted ratio (OR) for HbA1c ≥ 6.5% was1.28 (95% CI 1.07–1.55) (*p* = 0.008). In sensitivity analyses that included sample weights, the relationship between sodium intake and diabetes prevalence remained consistent and stable (Supplementary Table S3). The generalized additive model and the smoothed curve fit showed that sodium intake was positively associated with the prevalence of diabetes in participants. A linear association between sodium intake and the prevalence of diabetes was found (*p* = 0.003) (Figure 2). There was also a linear relationship between sodium intake and FBG ≥ 7 mmol/L (*p* = 0.009) and HBA1c ≥ 6.5% (*p* < 0.001) (Supplementary Figures S1, S2).

**Table 2:** Relationship between sodium intake and diabetes in different models among US adult.

sodium, mg	diabetes OR (95% CI), *p* value		
	Model I	Model II	Model III
Sodium intake (per 1,000 mg)	1.19 (1.06–1.34) 0.002	1.21 (1.07–1.36) 0.002	1.20 (1.07–1.35) 0.003
Q1	Reference	Reference	Reference
Q2	1.21 (0.93–1.59) 0.161	1.28 (0.97–1.69) 0.085	1.30 (0.98–1.72) 0.068
Q3	1.29 (0.94–1.77) 0.109	1.30 (0.94–1.79) 0.114	1.32 (0.96–1.83) 0.092
Q4	1.63 (1.04–2.35) 0.020	1.74 (1.14–2.66) 0.011	1.80 (1.17–2.76) 0.008
*p* for trend	0.027	0.021	0.016

CI, confidence interval; OR, odds ratio.

Model I was adjusted for age, sex, energy, protein, carbohydrate, fat.

Model II was adjusted for age, sex, energy, protein, carbohydrate, fat, race, poverty income ratio, BMI, education level.

Model III was adjusted for age, sex, race, poverty income ratio, BMI, education, energy, protein, carbohydrate, fat, UA, alcohol intake, eGFR, smoke exposure, total physical activity.

### Relationship between sodium intake and diabetes in subgroup

**Figure 3:**
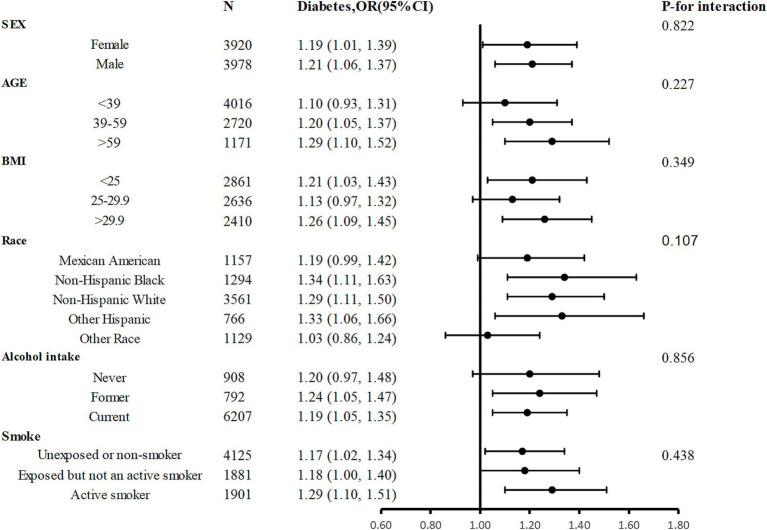
Stratified Analyses by Potential Modifiers of the Association between sodium intake and the prevalence of diabetes. Adjusted for age, sex, race, poverty income ratio, BMI, education, energy, protein, carbohydrate, fat, UA, alcohol intake, eGFR, smoke exposure, and total physical activity except the subgroup variable.

Participants were divided into several subgroups for a stratified analysis to assess the relationship between sodium intake and diabetes (Figure 3). Sodium intake was positively associated with prevalence of diabetes in different subgroups, with higher sodium intake associated with higher prevalence of diabetes, and this relationship remained stable across subgroups. None of the variables, including sex, age, race, BMI, smoking status, and drinking status significantly modified the association between sodium intake and the prevalence of diabetes (*p* for all interactions >0.05).

## Discussion

To our knowledge, this is the first large sample cross-sectional study to evaluate sodium intake and diabetes prevalence in non-hypertensive adults in the United States, emphasizing the association of a high sodium diet for diabetes prevalence. The present study shows that the proportion of diabetes is significantly large in the high sodium intake group. The linear association depicts that the prevalence of diabetes has a positive correlation with sodium intake. The risk of diabetes increased by 1.20 times for every 1 g increase in sodium. In addition, subgroup analysis showed a stable positive association between sodium intake and diabetes in all subgroups.

Suckling RJ et al. study showed that reducing sodium intake has no significant effect on fasting blood glucose, glycosylated hemoglobin, and insulin sensitivity in patients with diabetes and impaired glucose tolerance (17), In another 8-week randomized controlled trial, limiting salt intake had no significant effect on glucose and insulin metabolism in hypertensive patients (16). However, our study revealed a positive correlation between sodium intake and abnormal fasting blood glucose and glycohemoglobin. It is important to note that previous studies had limited sample sizes, different study populations, and the relatively short follow-up time could only reflect short-term conclusions, which may account for the disparity in their results compared to ours. Therefore, randomized studies with larger samples and longer follow-up times are needed to verify this conclusion.

A prospective study has observed that high sodium intake is the independent risk factor of type 2 diabetes by testing the urinary sodium of participants (18). And another Mendelian randomization study verified that urinary sodium is one of the 21 suggestive risk factors which were selected by 170 risk factors of type 2 diabetes (28). Similarly, Matti A. Vuori et al. has emphasized that high sodium intake is associated with an increased incidence of cardiovascular disease and diabetes (29). Furthermore, sodium intake adds to the risk of 2 diabetes was widely reported in Asia, such as China, Korea and Japan where pickles and sauces are popular (30–32). For the first time, our examination of a nationally representative non-institutional members in the United States corroborates previous research indicating a positive correlation between higher sodium intake and an increased risk of diabetes. Compared with previous studies, we utilized a complex sampling design and weighted our research results based on the recommendations of NHANES to prevent over-sampling. Furthermore, our study included a larger sample size and NHANES provided more detailed dietary data, such as the intake of total energy, sugar, protein and fat, as well as complete laboratory examination data, which may be potential risk factors for the onset of diabetes. We included these data and constructed different models to adjust these risk factors. All the abovementioned reasons may increase the reliability of our conclusions.

Compared to previous studies in the general population, our study included a specific group of people without high blood pressure, it is widely known that over sodium intake contributes to hypertension (33), and the results from a retrospective propensity score-matched cohort study in China have indicated that subjects with hypertension are more likely to develop diabetes with an 11.0% increased risk (34). In addition, the participants with hypertension usually were advised by physicians to a low-salt diet. Thus, in the present study, to eliminate the interference of hypertension factors on the diet and the diabetes outcome, we excluded the hypertension participants. Given the high co-incidence of hypertension and diabetes and the two diseases are risk factors for each other, the association between sodium intake and diabetes may be even stronger in people with hypertension.

Despite the lack of clarity on the pathophysiological connection between sodium intake and diabetes, several potential mechanisms could explain the association. Some studies have attributed the link to excess salt intake associated with increased consumption of sugar-containing beverages, obesity and high blood pressure, which are both risk factors for diabetes (18, 35–37), moreover, in diabetic animal models, a high-salt diet aggravated renal fibrosis and impaired fatty acid metabolism (38). However, our study excluded people with high blood pressure and adjusted for BMI, and the association remained. Previous studies have suggested that higher magnesium intake may improve glucose processing and insulin action, and reduce the risk of developing type 2 diabetes (39, 40), extracellular sodium level is related to intracellular magnesium transfer to extracellular and reabsorption of magnesium by distal convoluted tubules (41, 42). However, it is unclear how sodium intake affects a person’s blood glucose. Excessive salt consumption has been demonstrated to cause insulin resistance in some earlier research by decreasing insulin sensitivity, preventing insulin mRNA expression, weakening insulin signaling, and raising angiotensin II production (43–45). A prior animal study proved that the PPARδ/adiponectin/SGLT2 pathway may play an important role in the regulation of sodium and glucose homeostasis (46), through the activation of the adipose PPAR, high sodium intake raises adiponectin levels, which in turn downregulate renal SGLT2 and cause natriuresis and glycosuria. Miguel A Lanaspa et al. observed that high intake of salt activates the aldose reductase-fructokinase pathway in the liver and hypothalamus, resulting in endogenous fructose production with the development of leptin resistance and hyperphagia (47).

There are still some limitations of this study. First of all, it is not possible to infer causality due to the cross-sectional design, more prospective studies are needed to validate our findings. Second, in the evaluation of confounding factors, residual confounding effects due to measurement errors are inevitable. Third, NHANES surveys used interviews and questionnaires to obtain self-reported information, which may lead to inaccurate information and recall bias, however, a previous study found a strong link between self-reported dietary sodium intake and 24-h urine sodium measurement. Last, we were unable to separate the data by diabetes type 1 or type 2 status because the NHANES survey did not contain that specific data. But previous reports suggest that 8.5% of American adults have type 2 diabetes, while only 0.5% have type 1 diabetes, therefore our results may be more appropriate for type 2 diabetes (48). Despite these limitations, the study has a number of significant advantages. This study employed recent data that was quite representative because it was gathered from national representatives using well-designed protocols. What is more, we investigated the incidence of diabetes in a special population from another perspective by excluding the participants with hypertension, moreover, we have adjusted the correlation with sufficient covariates, and the results are independent and reliable. In addition, several sensitivity analyses were performed to examine the consistency of the results and to reveal and correct for possible polymorphisms.

Our results suggest that higher sodium intake is associated with an increased risk of diabetes in the population without hypertension, and for every 1,000 mg sodium intake increment, the risk of diabetes increased by 1.19 times. To sum up, we have provided the clue to the etiology of diabetes and further prospective research is needed to contribute recommendations for the primary prevention of diabetes in the US.

## Data availability statement

The datasets presented in this study can be found in online repositories. The names of the repository/repositories and accession number(s) can be found at: https://www.cdc.gov/nchs/nhanes/index.htm.

## Ethics statement

The NHANES is a series of cross-sectional, complex, multistage surveys conducted by the Centers for Disease Control (CDC) and Prevention of a nationally representative group of non-institutionalized members of the US population designed to provide health and nutrition data. Before taking part, participants provided written informed consent. The NCHS ethics review board reviewed and approved the collection of NHANES data. The studies were conducted in accordance with the local legislation and institutional requirements. The participants provided their written informed consent to participate in this study.

## Author contributions

LM, DW and YZ: protocol development and critical review and approval of the manuscript. LM: drafting of the manuscript, analysis, and interpretation of the data. YZ: analysis and interpretation of the data and approved the final version of the submitted manuscript. All authors contributed to the article and approved the submitted version.

## Conflict of interest

The authors declare that the research was conducted in the absence of any commercial or financial relationships that could be construed as a potential conflict of interest.

## Publisher’s note

All claims expressed in this article are solely those of the authors and do not necessarily represent those of their affiliated organizations, or those of the publisher, the editors and the reviewers. Any product that may be evaluated in this article, or claim that may be made by its manufacturer, is not guaranteed or endorsed by the publisher.
