# Supplementary material for: Association of sodium intake with diabetes in adults without hypertension: evidence from the National Health and Nutrition Examination Survey 2009–2018
Source: Front Public Health. 2023 Aug 31;11:1118364. doi: 10.3389/fpubh.2023.1118364 (PMC10506081; doi:10.3389/fpubh.2023.1118364)
Supplement: Supplementary file 3 [file Table_3.DOC]

**Table s3.**Association between sodium intake and diabetes in different models among US adult(weighted)

| sodium, mg | diabetes OR (95% CI), P value | | |
| --- | --- | --- | --- |
|  | Model Ⅰ | Model Ⅱ | Model Ⅲ |
| Sodium intake(per 1000mg) | 1.22(1.04-1.44)0.016 | 1.21(1.02-1.42)0.030 | 1.21(1.03-1.43)0.030 |
| Q1 | Reference | Reference | Reference |
| Q2 | 1.27(0.82-1.96)0.296 | 1.29(0.82-2.04)0.266 | 1.31(0.83-2.06)0.253 |
| Q3 | 1.46(0.89-2.38)0.134 | 1.40(0.84-2.31)0.191 | 1.42(0.86-2.33)0.173 |
| Q4 | 2.00(1.05-3.83)0.040 | 1.95(0.99-3.85)0.053 | 2.05(1.05-4.01)0.041 |
| *p* for trend | 0.043 | 0.066 | 0.016 |
| CI=confidence interval. OR=odds ratio.  Model I was adjusted for age, sex, energy, protein, carbohydrate, fat  Model II was adjusted for age, sex, energy, protein, carbohydrate, fat, race, poverty income ratio, BMI, education level  Model III was adjusted for age, sex, race, poverty income ratio, BMI, education, energy, protein, carbohydrate, fat, UA, alcohol intake, eGFR, smoke exposure, total physical activity | | | |
