# Supplementary figures and images for: Association of sodium intake with diabetes in adults without hypertension: evidence from the National Health and Nutrition Examination Survey 2009–2018
Source: Front Public Health. 2023 Aug 31;11:1118364. doi: 10.3389/fpubh.2023.1118364 (PMC10506081; doi:10.3389/fpubh.2023.1118364)

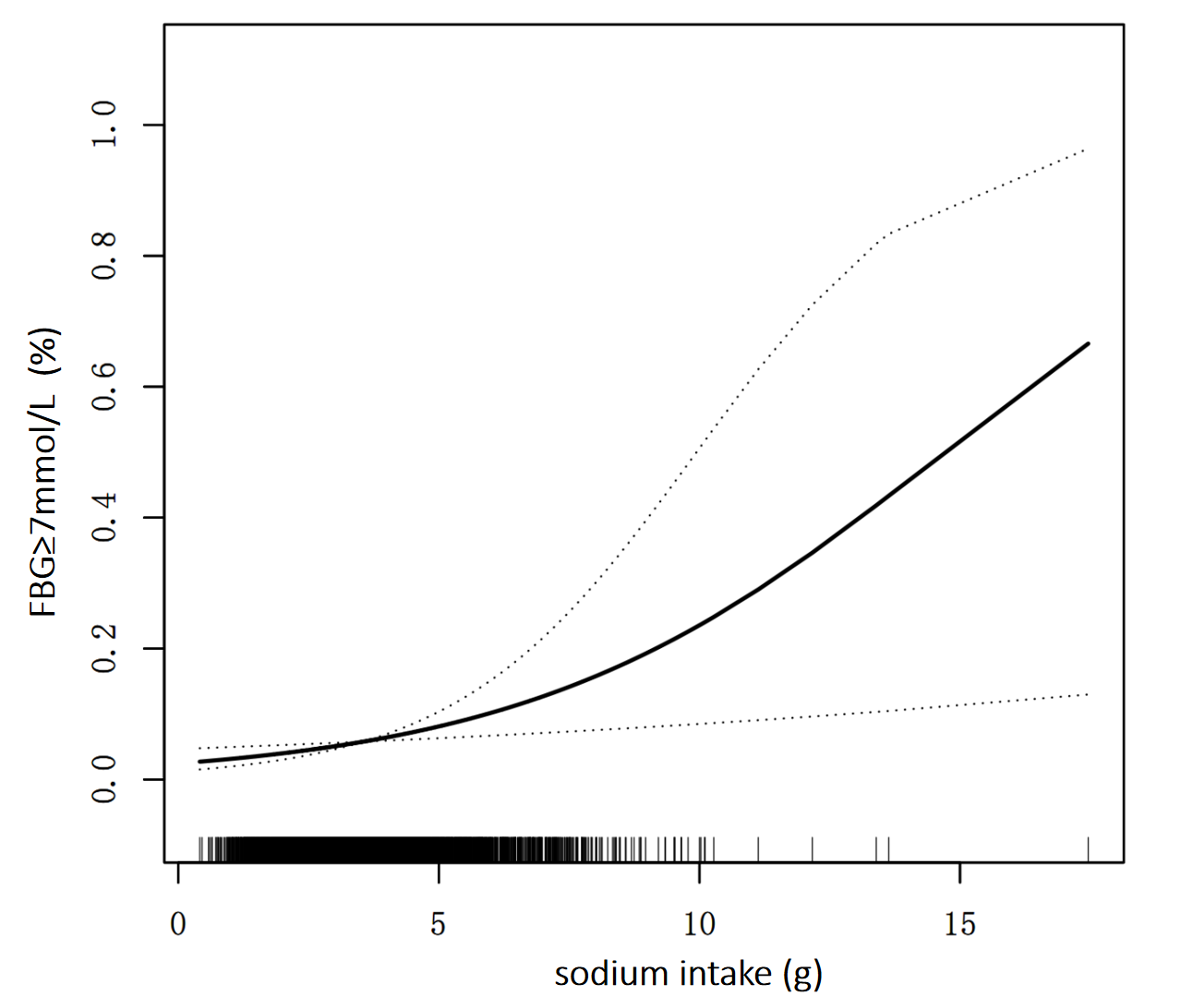

Supplement: Supplementary file 4 [file Image_1.PNG]

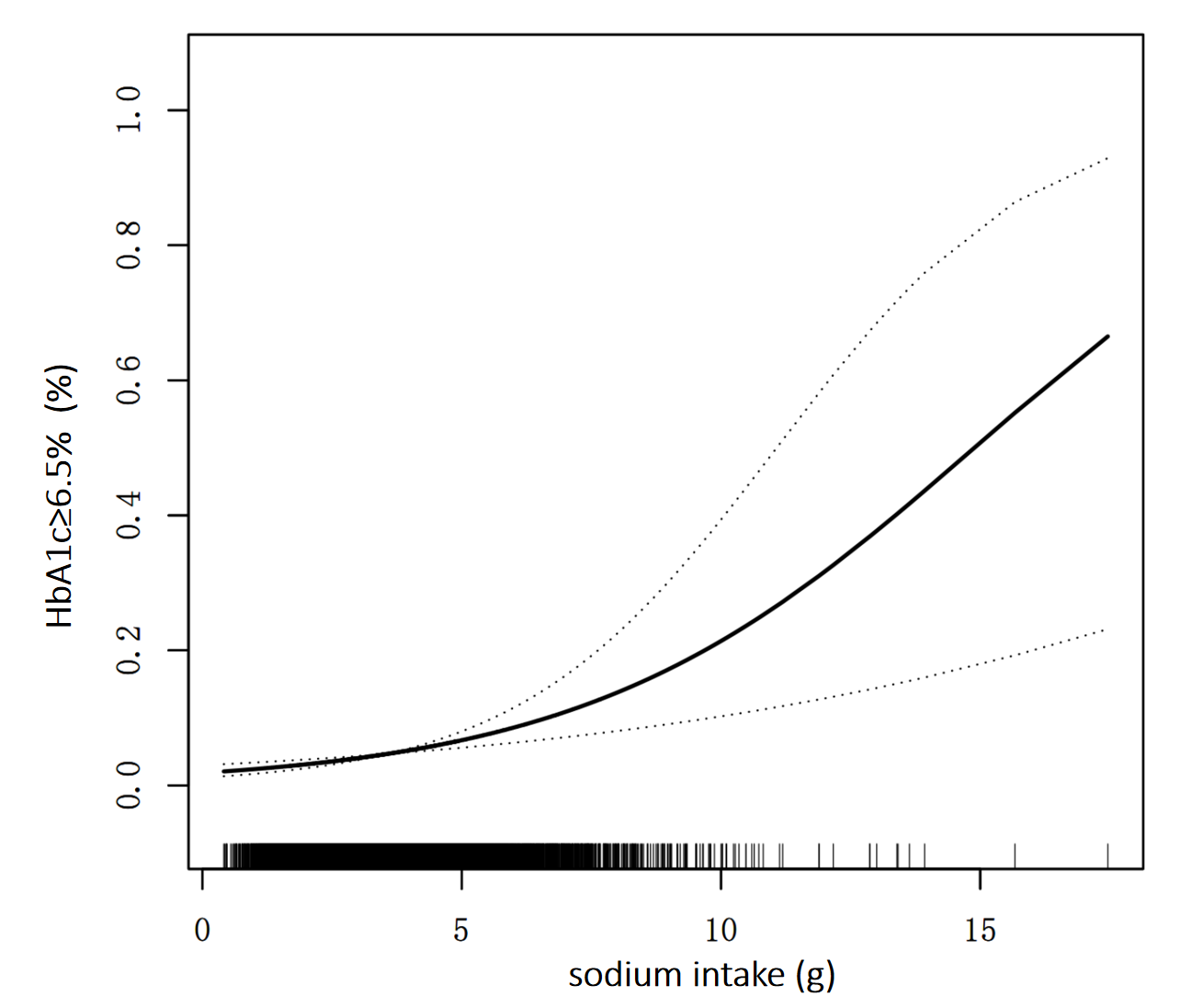

Supplement: Supplementary file 5 [file Image_2.PNG]
